# Structural Manipulation of Spin Excitations in a Molecular Junction

**DOI:** 10.1021/acs.nanolett.4c04075

**Published:** 2024-10-30

**Authors:** Maximilian Kögler, Nicolas Néel, Laurent Limot, Jörg Kröger

**Affiliations:** † Institut für Physik, Technische Universität Ilmenau, D-98693 Ilmenau, Germany; ‡ Institut de Physique et Chimie des Matériaux de Strasbourg, Université de Strasbourg, F-67000 Strasbourg, France

**Keywords:** nickelocene, spin excitation, atomic manipulation, inelastic electron tunneling spectroscopy, scanning tunneling microscopy

## Abstract

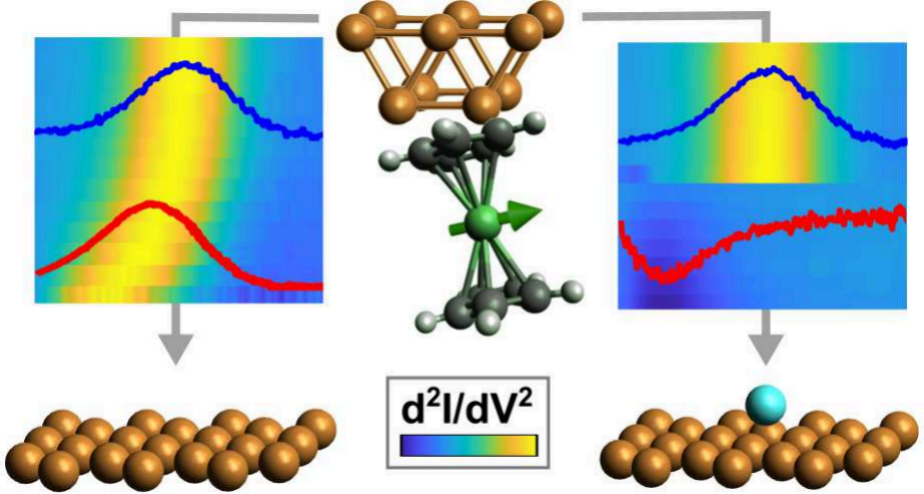

Single metallocene molecules act as sensitive spin detectors when decorating the probe of a scanning tunneling microscope (STM). However, the impact of the atomic-scale electrode details on the molecular spin state has remained elusive to date. Here, a nickelocene (Nc) STM junction is manipulated in an atomwise manner showing clearly the dependence of the spin excitation spectrum on the anchoring of Nc to Cu(111), a Cu monomer, and trimer. Moreover, while the spin state of the same Nc tip is a triplet with tunable spin excitation energies upon contacting the surface, it transitions to a Kondo-screened doublet on a Cu atom. Notably, the nontrivial magnetic exchange interaction of the molecular spin with the electron continuum of the substrate determines the spectral line shape of the spin excitations.

Single magnetic molecules are promising for a variety of applications in, for example, quantum storage,^1,2^ quantum computing,^3−5^ molecular spintronics,^6−10^ and spin sensors,^11−15^ because they often exhibit high spin–orbit coupling that provides magnetic anisotropy, which in turn gives rise to preferred spin directions without an external magnetic field. Moreover, molecular magnetism can be tailored by, for example, hybridization with the environment,^16−19^ charge transfer,^7,20,21^ or structural relaxations caused by mechanical stress.^22−24^ Apart from these appealing aspects from a nanotechnological perspective, the coupling of spin excitations of a single magnetic impurity to the electron continuum of a substrate represents an important model system for the understanding of fundamental topics in quantum physics, such as spin-polarized electron transport,^25−34^ magnetic exchange interaction,^35−38^ spin textures at surfaces,^39−43^ and electron correlations.^44−49^

The scanning tunneling microscope (STM) appears to be particularly suitable for exploring quantum excitations in controlled environments, a favorable opportunity that results from the combined capabilities of imaging and manipulating matter at the atomic scale together with highly resolved spectroscopy.^50^ Furthermore, the intentional termination of the tip apex with single atoms or molecules extends the control of the junction geometry and enables additional functionality such as spin sensitivity.

Very recently, nickelocene (NiCp_2_ (Nc), Cp: cyclopentadienyl, Figure 1a) has been shown to retain its gas-phase spin triplet (spin quantum number *S* = 1) ground state upon adsorption on surfaces as well as on the apex of an STM tip and to act as a molecular spin detector.^20,51,52^ The frontier orbitals of Nc are mostly composed of Ni *d*-orbitals with an admixture of C *p*-states. The spin–orbit interaction induces a uniaxial magnetic anisotropy  with an easy plane parallel to the Cp moieties.^20^ The spin excitation energy *D* separates the ground state with a spin magnetic quantum number *M*_*S*_ = 0 from the degenerate excited spin states with *M*_*S*_ = ±1 (Figure 1b).

**Figure 1:**
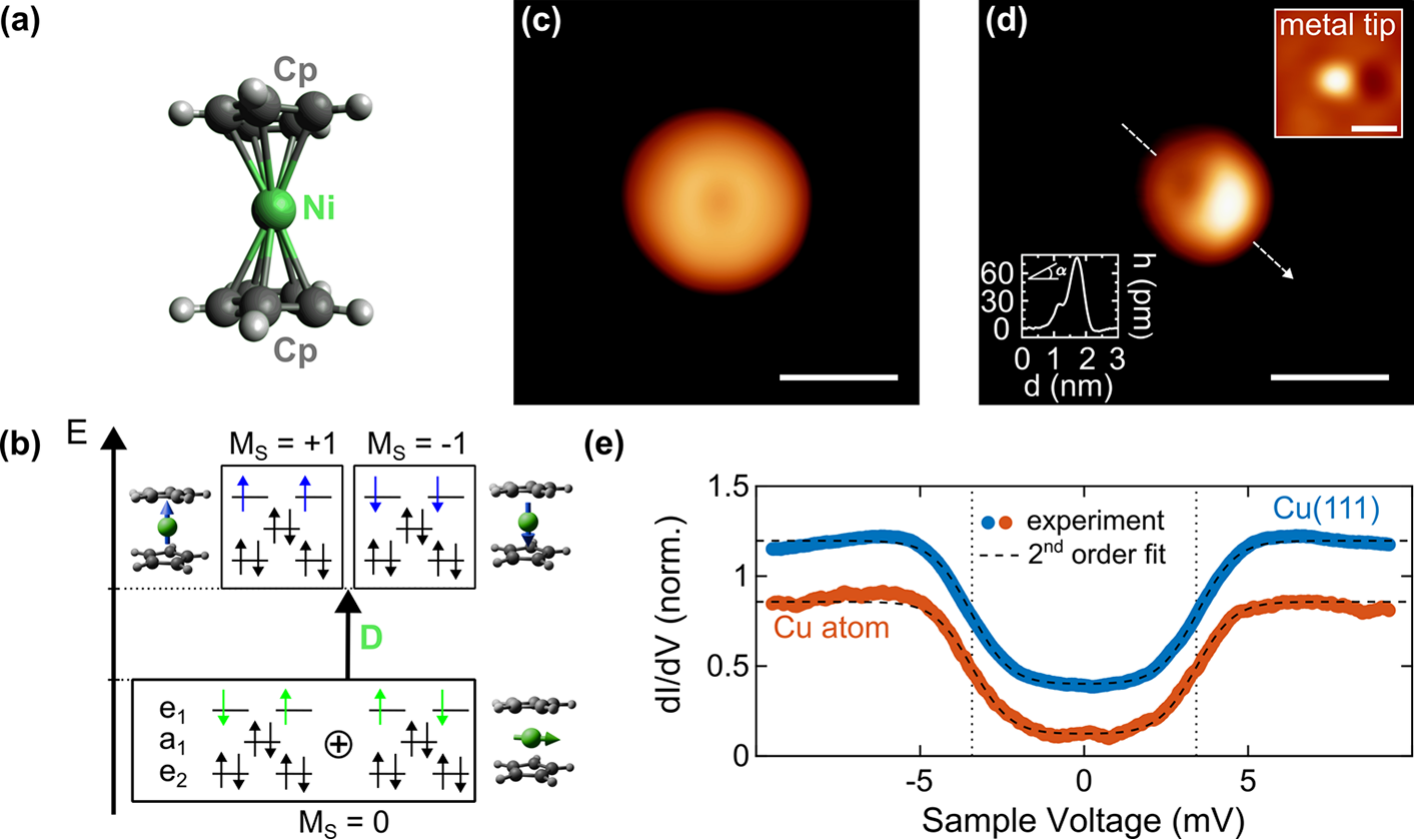
Spin excitations of Nc. (a) *D*_5*h*_ symmetry of free-Nc molecular backbone with two parallel Cp planes sandwiching the Ni ion. (b) Ligand-field-induced lifting of the Ni *d*-orbital degeneracy resulting in the ground state (*M*_*S*_ = 0; 4 electrons in the low-energy *e*_2_-terms (*d*_*x*^2^ – *y*^2^_, *d*_*xy*_); 2 electrons on the middle-energy *a*_1_-term (d_*z*_^_2_^); 1 electron in the *e*_1_-terms (*d*_*xz*_, *d*_*yz*_) each) and the excited state (*M*_*S*_ = ±1), which are separated by the spin excitation energy *D* > 0. (c) Metal-tip STM image of Nc residing at a surface defect of Cu(111) (sample voltage: −40 mV; tunneling current: 20 pA) revealing the parallel orientation of the top Cp plane to Cu(111). (d) STM image of a single Cu adatom recorded with a Nc tip (−200 mV, 20 pA). Insets: metal-tip STM image of the adatom (top) and cross-sectional profile acquired along the dashed line in (d) (bottom). Scale bars in all STM images indicate 1 nm. (e) Spectra of d*I*/d*V* acquired with an Nc tip atop Cu(111) (top) and a Cu adatom (bottom). Feedback loop parameters: −10 mV and 50 pA (surface) and 20 pA (adatom). The spectra are normalized to their respective maximum and are vertically offset. Dashed lines are fits to the data using a second-order dynamical exchange scattering model (see the text). Dotted lines mark the spin excitation flanks at voltages ±*D*/e with *D* resulting from the second-order fit.

The exchange interaction between the Nc tip and various magnetic adsorbates on surfaces has successfully been probed by the sensitive response of the Nc spin excitation spectrum.^51−54^ In addition, the spin state of Nc at the STM tip can be manipulated by bringing the Nc tip close to the surface.^16,17^ The transition from *S* = 1 to *S* = 1/2 is reflected by the abrupt change of the spin excitation gap in spectroscopy of the differential conductance (d*I*/d*V*, *I*: current, *V*: sample voltage) to a zero-voltage resonance that is assigned to the Kondo effect.^16^ A gradual closing of the spin excitation gap was likewise observed,^17^ albeit less frequently.

While Nc-decorated probes have been demonstrated to act as sensitive spin sensors when nearly in contact with surfaces, the impact of the atomic structure of the electrodes (tip and surface) on the Nc spin states has experimentally not been addressed so far. Therefore, the dependence of the spin excitation spectrum is explored here on the specific atomic environment of Nc adsorbed on Cu(111), a single Cu atom, and an artificially fabricated linear Cu_3_ cluster. In addition, variations of the spin excitation spectra for a single Nc decorating a blunt STM tip approaching the surface and an adsorbed atom (adatom) are compared. The experimental observations are manifold. Spin excitation energies depend on the atomic sharpness of the electrode to which Nc is attached, with a maximum energy attained for the single-atom-terminated electrode. Moreover, for the approach to Cu(111) the gradual decrease of the spin excitation energies and the eventual evolution of a zero-voltage resonance in d*I*/d*V* spectroscopy at Nc–surface contact is observed. In contrast, the experiments with the same Nc tip on the adatom lead to the sudden disappearance of the spin excitation gap for the benefit of a d*I*/d*V* zero-voltage resonance. These findings furthermore unveil the hitherto elusive mechanism for gradual and abrupt changes in Nc spin excitation spectra.^17^

Stable imaging of single Nc molecules with STM was hampered by perpetual lateral translations of the molecules in the presence of the tip, which is indicative of a weakly corrugated adsorption potential for Nc on Cu(111). Only at surface defect sites, e.g., bulk-segregated impurities or lattice defects, does Nc reside in a manner that enables stable STM imaging, as shown in Figure 1c. The broad ring with a central depression is assigned to the top Cp moiety of an upright adsorbed Nc, in agreement with observations from Ag(110)^51^ and Cu(100).^16,17,20,52,53,55^ Such Nc molecules can readily be transferred to the tip by reducing the separation between the tip and the Nc center at a negative sample voltage (−1 mV, Supporting Information, Section S1). The successful transfer can be verified by imaging an atom adsorbed on the surface, which represents an elegant method to geometrically characterize the atomic-scale details of the tip.^56^ A Cu adatom appears with a ringlike structure in STM images acquired with the Nc-decorated tip (Figure 1d). The asymmetric brightness of the ring and the off-center depression indicate a tilted axis of the Nc molecule terminating the tip, which is estimated as ≈6° with respect to the surface normal (Supporting Information, Section S2).

In addition to imaging, spectroscopy of d*I*/d*V* with the Nc-terminated tip was performed on Cu(111) (top spectrum in Figure 1e) and atop a Cu adatom (bottom). Both spectral data were recorded in the far tunneling range of tip–surface distances and exhibit a virtually identical gap with flanks symmetrically positioned around zero sample voltage, which are attributed to the spin excitation from *M*_*S*_ = 0 to *M*_*S*_ = ±1, i.e., to the spin flip from the horizontal to the vertical spin orientation. Matching the data with *f*(*V*) = σ_0_·[Ξ(*eV* – *D*, *T*) + Ξ(−*eV* – *D*, *T*)] + σ_el_, which results from the second-order scattering model^57^ and where Ξ is the temperature-broadened step function^58^ with σ_0_, *D*, *T*, and σ_el_ serving as fit parameters, yields *D* = 3.7 ± 0.2 meV, which is in agreement with earlier results.^20^ The values represent the arithmetic mean and standard deviation obtained from 10 different Nc tips.

The scattering of *D* is assigned to the atomic-scale environment in which Nc is embedded, which is an important finding of the studies presented here and which helps elucidate the evolution of the Nc spin excitation spectra upon contact, to be discussed below. To see the impact of the atomic electrode geometry on the spin excitation energy, Nc-decorated tips were mimicked by Nc molecules adsorbed on a single Cu atom on Cu(111), on an assembled linear Cu_3_ cluster, and on Cu(111), which model atomically sharp, less sharp, and blunt tip configurations, respectively. Single Cu atoms were transferred from the tip to the surface,^59^ while Cu_3_ chains were assembled by atom manipulation (Supporting Information, Section S3 and Figure S1). In agreement with previous reports,^60,61^ Cu dimers and compact Cu trimers were unstable at the experimental temperature.

The associated spin excitation spectra are depicted in Figure 2a–c and show that the maximum *D* is obtained for Nc residing on the adatom (*D*_max_ ≈ 4.14 meV). The excitation energy decreases to *D* ≈ 3.31 meV for Nc on linear Cu_3_ and reaches its minimum of *D*_min_ ≈ 3.21 meV on pristine Cu(111). It is noteworthy that spectroscopy of Nc on clean Cu(111) was feasible only in the far tunneling range due to the aforementioned junction instabilities, while molecules anchored at surface defects (Figure 1c) did not indicate spin excitation signatures. Therefore, the spin excitation energy of Nc in these different contact junctions can be varied by nearly 1 meV, which corresponds to a modification of *D* by ≈30%.

**Figure 2:**
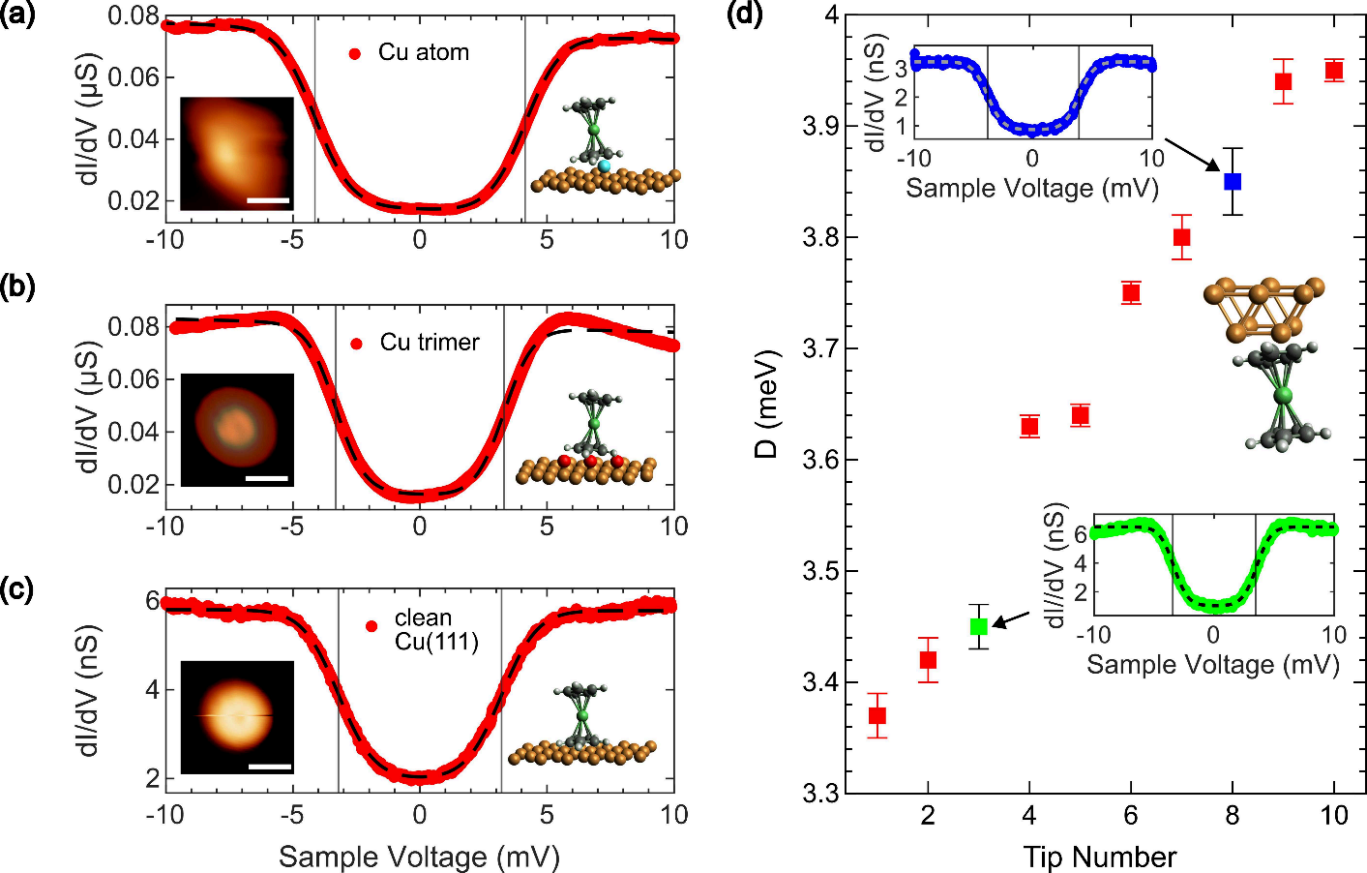
Influence of the electrode structure on the Nc spin excitation energy. (a) Spectrum of d*I*/d*V* acquired with a pristine Cu tip atop the center of a single Nc molecule adsorbed on a single Cu atom on Cu(111) (feedback loop parameters: 10 mV, 500 pA). Insets: STM image of adsorbed Nc (80 mV, 20 pA) and sketch of the suggested adsorption geometry. (b) As (a) for Nc adsorbed on a linear Cu_3_ cluster (10 mV, 600 pA). (c) As (a) for Nc adsorbed on bare Cu(111) (10 mV, 50 pA). The scale bars in all STM images indicate 1 nm. (d) Collection of *D* extracted from spin excitation spectra acquired in the far tunneling range (10 mV, 30 to 100 pA) with 10 Nc-decorated tips atop Cu(111). The uncertainty margins mark variations of the second-order fit algorithm. Insets: exemplary d*I*/d*V* spectra associated with the marked data points and a sketch of suggested Nc decoration of a blunt tip. The vertical lines in all spectra mark *V* = ±*D*/*e*, with *D* resulting from fits (dashed lines) to the d*I*/d*V* data within the second-order scattering model (Supporting Information, Section S7 and Figure S6).

The entire set of 10 Nc tips that were prepared for the experiments gave rise to spin excitation spectra with energies ranging between *D*_min_ and *D*_max_ (Figure 2d). In particular, based on these results the Nc tip used in the experiments discussed next is proposed to belong to the class of rather blunt tips because the extracted *D* ≈ 3.45 meV is close to *D*_min_ (bottom inset to Figure 2d).

In the next step, the evolution of the spin excitation spectra atop Cu(111) and the Cu adatom was explored with decreasing separation between the Nc tip and the surface. The collection of all d^2^*I*/d*V*^2^ spectra for *V* ≥ 0 as a function of tip excursion *Δz* on Cu(111) is depicted in Figure 3a. The spectra in the far tunneling range are dominated by a peak at 3.7 mV, which corresponds to the spin excitation of Nc (Figure 1e). With increasing *Δz*, i.e., with decreasing tip–surface distance, the spin excitation signature shifts to lower sample voltages in a gradual manner, similar to observations on Cu(100).^17^ At the largest tip excursions used in the experiments, the excitation peak appears at 1.7 mV.

**Figure 3:**
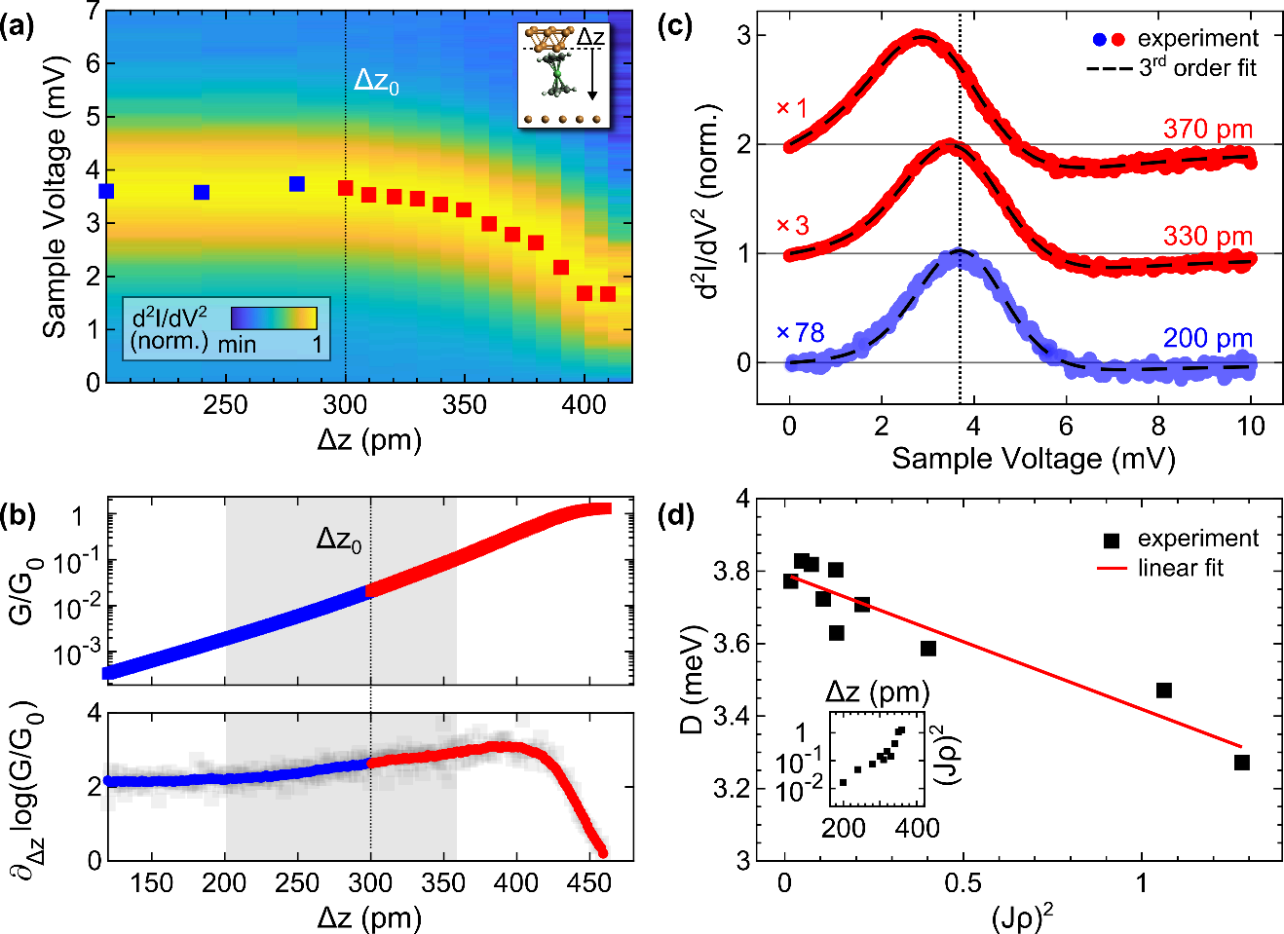
Approach of an Nc tip to Cu(111). (a) Collection of d^2^*I*/d*V*^2^ spectra as a function of tip excursion *Δz*. The spectra are normalized to their respective maxima. Symbols mark the d^2^*I*/d*V*^2^ peak positions. At *Δz*_0_ ≈ 300 pm, the shift of the spin excitation peaks toward 0 V becomes detectable. Feedback loop parameters defining tip excursion *Δz* = 0: 10 mV, 20 pA. (b) Top: variation of *G*/*G*_0_ at 10 mV as a function of tip approach (from low to high *Δz*) on a logarithmic scale. Bottom: derivative of log(*G*/*G*_0_) with respect to *Δz*. The shaded area marks the *Δz* region where weak deviations of *G*(*Δz*) from a uniform exponential behavior occur. (c) Selected d^2^*I*/d*V*^2^ spectra (dots) at the indicated *Δz* for illustrating the line shape analysis within the third-order dynamical scattering model (dashed lines). The vertical line marks the position of the spin excitation peak in the far tunneling spectrum. The spectra are vertically offset, with the horizontal lines marking the respective zeros of d^2^*I*/d*V*^2^. (d) Evolution of *D* with (*J*ϱ)^2^ (squares) as extracted from the third-order fits for 200 pm ≤ *Δz* ≤ 360 pm. The solid line is a linear fit to the data. Inset: variation of (*J*ϱ)^2^ with *Δz*.

To clearly see the collapse of the tunneling barrier at contact, traces of the junction conductance *G* = *I*/*V* are useful.^62,63^Figure 3b shows *G* = *G*(*Δz*) in units of the conductance quantum *G*_0_ = 2 *e*^2^/*h* (*e*: elementary charge; *h*: Planck constant) where the strongest deviation from a uniform exponential variation occurs starting from *Δz* ≳ 420 pm. The conductance trace then levels off and reaches a nearly constant value of 1.3 *G*_0_, which is comparable to other single-molecule contacts.^62−66^ This range of tip excursions is, therefore, associated with the Nc–Cu(111) contact. Importantly, weak deviations from a uniform exponential *G*(*Δz*) behavior are already observed for tip excursions indicated by the gray rectangle in Figure 3b (200 pm ≤ *Δz* ≤ 360 pm). These changes are more clearly identified in the plot of derivative ∂ log(*G*/*G*_0_)/∂Δ*z* in the bottom panel of Figure 3b. They are compatible with the shift of the spin excitation signature to lower bias voltage (Supporting Information, Section S4 and Figure S2). Therefore, in this range of tip excursions, relaxations such as the deformation of the Nc backbone are negligible.

The evolution of the spin excitation can be understood from careful analysis of its line shape in d^2^*I*/d*V*^2^ spectroscopy. Figure 3c shows three exemplary spectra at different *Δz*. Besides the peak shift to lower voltage with increasing *Δz*, the high-voltage tail of the peak exhibits a remarkable evolution. In the far tunneling range of *Δz* (bottom spectrum in Figure 3c), the tail is virtually horizontal. With increasing *Δz*, i.e., with increasing *G*, the tail evolves into a shallow and broad minimum. The second-order tunneling model, which proved successful for describing the spin excitation spectra in the tunneling range (dashed lines in Figure 1e), is therefore no longer applicable to the description of the spectral data at increased *G* (Supporting Information, Section S5 and Figure S3). Rather, higher-order physical effects have to be considered for explaining the actual variation of the d^2^*I*/d*V*^2^ signal with the voltage.

A possible rationale for the line shape evolution is the coupling of the Nc spin excitation to the substrate electron continuum via Kondo exchange interaction.^67^ It is mediated by the hybridization of the magnetic impurity with the electron gas of the metal, which in the experiments is tuned by the proximity of the Nc-terminated tip to the surface. Including the Kondo exchange interaction into the dynamical scattering model via the second-order Born approximation gives rise to logarithmic contributions in d*I*/d*V* at *V* = ±*D*/e scaling with *Jϱ* (*J*: Kondo exchange energy, ϱ: sample density of states at the Fermi energy). This extended third-order tunneling model^57,68,69^ matches the experimental data very well (Supporting Information, Section S5 and Figure S4). Moreover, the experimentally extracted *D* for 200 pm ≤ *Δz* ≤ 360 pm follows the predicted behavior, *D* = *D*_0_ – *c*(*J*ϱ)^2^ (*D*_0_: spin excitation energy without Kondo exchange coupling, *c*: constant factor containing the bandwidth of the relevant substrate electrons and the temperature),^18,70^ as demonstrated in Figure 3d. Therefore, in agreement with observations and conclusions reported from Cu(100),^17^ the gradual closing of the spin excitation gap is assigned to the increasing Kondo exchange interaction with the eventual Kondo screening of the *S* = 1 impurity at contact.

In the case of Cu(100) the predicted variation of *D* with *Jϱ* is likewise applicable (Supporting Information, Section S6 and Figure S5). Because the predicted behavior of *D* with *Jϱ* is well verified by the experimental data, a possible decrease of *D* due to junction relaxations or charge transfer is surmised here to play a minor role.

The same Nc tip that was used for the experiments on Cu(111) then likewise served as the probe for the Cu adatom (Figure 4). The Nc spin excitation occurs at a voltage (±3.7 mV) similar to that on the pristine surface. However, its evolution with increasing tip excursion is markedly different (Figure 4a). Upon entering the contact range, which is signaled by the deviation of the junction conductance from its uniform exponential increase (**1** in Figure 4b), the excitation peak disappears and a dip occurs instead in d^2^*I*/d*V*^2^ spectra.

**Figure 4:**
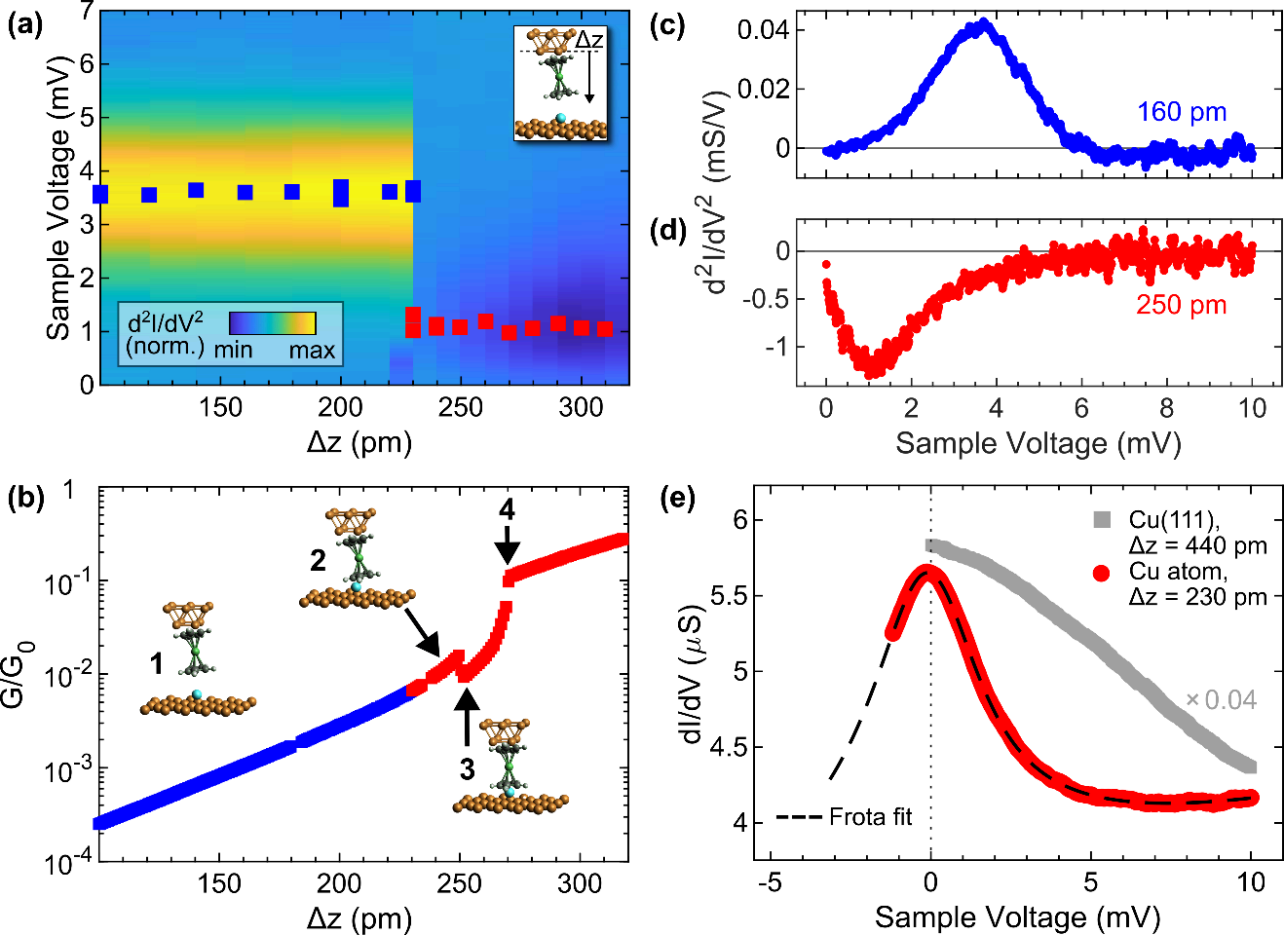
Approach of the same Nc tip as that in Figure 3 to a Cu adatom on Cu(111). (a) Collection of d^2^*I*/d*V*^2^ spectra as a function of tip excursion *Δz*. Symbols mark peak and dip positions. The spectra are normalized to the respective junction current at 10 mV. Feedback loop parameters defining *Δz* = 0: 10 mV, 20 pA. (b) Variation of the junction conductance at 10 mV with *Δz*. Insets: illustrations of suggested junction geometries in the tunneling (**1**) and contact (**2**–**4**) ranges. (c) Exemplary d^2^*I*/d*V*^2^ spectrum in the tunneling range (*Δz* = 160 pm). (d) As (c) for the contact range (*Δz* = 250 pm). (e) Integrated d^2^*I*/d*V*^2^ spectrum (*Δz* = 230 pm, dots) for an extended voltage range that was fit with a Frota function (dashed line). Gray symbols depict the d*I*/d*V* data obtained from the Nc–Cu(111) contact.

A typical d^2^*I*/d*V*^2^ spectrum in the tunneling range is depicted in Figure 4c. It bears a resemblance to the spectrum recorded atop Cu(111) (Figure 3c). At contact, however, a dip occurs in the spectroscopic data (Figure 4d), whose origin will be clarified in the following. To this end, the voltage range was extended to −2 mV. Below, junction instabilities occurred that hampered spectroscopy of the junction current. A representative contact spectrum with the extended voltage range was numerically integrated, giving rise to a peak centered at nearly 0 V (Figure 4e). Compared with integrated d^2^*I*/d*V*^2^ spectra of Cu(111) at comparable tip–surface distance (gray line in Figure 4e), the line width of the peak for the adatom is considerably narrower. The Frota line shape,
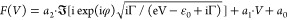
(φ: asymmetry factor, Γ: resonance width, ε_0_: resonance energy, i^2^ = −1), best matches the adatom data (dashed line in Figure 4e). To account for thermal and voltage modulation broadening, the Frota peak was additionally convoluted with the associated functions.^71,72^ Therefore, and because of previous findings on Cu(100),^16,17^ it is reasonable to assume that the peak in d*I*/d*V* at contact represents the Abrikosov–Suhl (AS) resonance, which signals the Kondo effect of an *S* = 1/2 impurity.^73^ In this case, the Kondo temperature becomes^74,75^*T*_K_ = Γ/(1.455*k*_B_) = 8 ± 1 K (*k*_B_: Boltzmann constant), and the resonance energy ε_0_ essentially vanishes. Based on the experimental results, the abrupt change in the spectral data is related to a transition of the Nc spin state from *S* = 1 to *S* = 1/2. The former requires energy *D* for a spin flip and, therefore, prevents the Kondo effect,^76^ while the latter exhibits degenerate electron spin levels^16^ where Kondo spin flip processes occur with vanishing energy cost.

The *G*(*Δz*) trace (Figure 4b) contains valuable clues about the mechanism underlying the spin transition. Unlike the behavior observed from approaching Cu(111), the *G*(*Δz*) data for the adatom exhibits kinks. The accelerated increase of *G*(*Δz*) (left arrow in Figure 4b) is likely related to the verge of the Nc–adatom contact, which is preferably formed with the Cp C–C bond (**2** in Figure 4b).^16,17^ The subsequent decrease in conductance (middle arrow in Figure 4b) may be associated with the centering of the Cu adatom in the Cp ring of Nc, which is achieved by a straightening of the molecule in the junction (**3** in Figure 4b). A similar scenario was previously put forward for a Cu adatom on Cu(100) mimicking a single-atom tip in simulations.^16^ Further increase of *Δz* is accompanied by a rapid rise of *G* (**3** to **4** in Figure 4b), which transitions into a uniform increase at **4**. Presumably, at **4** an Nc–adatom contact configuration is reached that is not subject to strong relaxations with increasing *Δz*. This *G* region reliably signaled the spin transition, i.e., the presence of the zero-voltage resonance in d*I*/d*V* spectra (Figure 4e). Importantly, imaging the adatom and probing the spin excitation gap of the Nc tip atop the bare surface ensured the structural integrity of the junction. These control experiments were intermediately performed during the entire approach experiment.

The remaining question concerns the experimentally observed transition behavior of the spin excitation spectra, i.e., the gradual-versus-abrupt evolution of the excitation gap into a peak in d*I*/d*V* spectroscopy. The experiments reported here help clarify the situation because the contact to the adatom with a blunt Nc-terminated tip is accompanied by an abrupt transition of the Nc spin excitation gap into a d*I*/d*V* zero-voltage peak that is similar to the observations reported from Cu(100)^16,17^ in the absence of adatoms but, presumably, with atomically sharp Nc-terminated tips. When the adatom is supposedly centered in the Cp ring upon straightening of Nc in the junction (**3** in Figure 4b), the localized and protruding adatom orbitals can penetrate the Cp ring and hybridize with the Ni *d*-states. The previously robust spin state *S* = 1 is no longer protected by the C *p*-orbitals of the Cp moiety and transitions to *S* = 1/2. The hybridization of Nc orbitals and metal states was indeed demonstrated in density-functional simulations to impact the molecular magnetic moment and the spin excitation energy.^77,78^ It is conjectured now that the straight Nc–atom junction may be equivalently formed at the tip apex. In this suggested scenario, the Cp group of Nc centers the Cu apex atom of the tip upon contact to the otherwise atomically flat substrate surface. Previous calculations assuming a single-atom-terminated tip revealed this molecular relaxation at the tip in contact geometries, which entailed a decrease of the Ni magnetic moment from 1 μ_B_ to 0.5 μ_B_ (μ_B_: Bohr magneton).^16^ Consequently, the presence of the adatom in the single-Nc junction is required for the abrupt closing of the spectroscopic spin excitation gap, while its gradual disappearance is related to atomically flat electrodes.

In conclusion, the presented findings show that the spin excitation energy of a single magnetic molecule is dependent on its atomic environment. Atomic-scale engineering of the electrode geometry the magnetic molecule is attached to allows tuning of the excitation energies by nearly 30%. The hitherto elusive mechanism underlying the gradual and abrupt closure of the spin excitation energy gap has been related to flat and atomically sharp electrodes attached to the molecule. The results of this work are important for spintronics devices that rely on the stable spin structure, e.g., in data storage applications. From a quantum-physics and quantum-chemistry perspective, the work unveils that a spin state protected by a molecular matrix can be altered by atomic orbitals protruding into the matrix and hybridizing with the spin-carrying energy levels.

## Methods

The experiments were performed with an STM operated in an ultrahigh vacuum (10^–9^ Pa) and at low temperature (5 K). Surfaces of Cu(111) were cleaned and prepared by Ar^+^ ion bombardment and annealing. The clean surface was exposed at a temperature between 5 and 20 K to Nc molecules sublimated from a powder (purity: ≥98.5%) in a ceramic crucible held at room temperature. During Nc deposition the pressure rose to 5 × 10^–7^ Pa. A coverage of approximately 20 Nc molecules per 80 nm × 80 nm was achieved for an evaporation time of 5 min. A chemically edged W wire (purity: 99.95%, diameter: 50 μm) served as the tip material. Field emission on and repeated indentations into the Cu surface presumably led to the coating of the tip apex with the substrate material. Single-atom transfer from the tip to the sample gave rise to particularly sharp and stable probes.^30,59,62,63^ Topographic data were acquired in the constant-current mode with the bias voltage applied to the sample and were further processed with WSxM.^79^ Spectroscopy of d*I*/d*V* and d^2^*I*/d*V*^2^ proceeded via the sinusoidal modulation (250 μV, 726 Hz for d*I*/d*V* and 363 Hz for d^2^*I*/d*V*^2^) of the dc sample voltage and measuring, respectively, the first and second harmonics of the ac current response of the tunneling junction with a lock-in amplifier.

## Data Availability

The authors declare that relevant data supporting the findings of this study are available on request.

## Supporting Information Available

Tip termination, tilt angle of Nc-decorated tip, fabrication of molecule–cluster assemblies, junction relaxations, dynamical scattering models, Kondo exchange renormalization, third-order analysis applied to Nc tips in the far tunneling range (PDF)

## Notes

During the review process we became aware of a theoretical study by Blesio et al.^80^ proposing the preservation of *S* = 1 for both the gradual and the abrupt change of the spin excitation spectra. It involves a topological quantum phase transition between a Fermi liquid and a non-Landau Fermi liquid. The experimental verification of this scenario requires very low temperatures.
