# Supplementary material for: Structural Manipulation of Spin Excitations in a Molecular Junction
Source: Nano Lett. 2024 Oct 30;24(45):14355–62. doi: 10.1021/acs.nanolett.4c04075 (PMC11566111; doi:10.1021/acs.nanolett.4c04075)
Supplement: Supplementary file 1 — nl4c04075_si_001.pdf [file nl4c04075_si_001.pdf]

Supporting Information

Structural Manipulation of Spin Excitations in a Molecular Junction

Maximilian Kögler,*,† Nicolas Néel,† Laurent Limot,‡ and Jörg Kröger†

†*Institut für Physik, Technische Universität Ilmenau, D-98693 Ilmenau, Germany*

‡*Institut de Physique et Chimie des Matériaux de Strasbourg, Université de Strasbourg, F-67000 Strasbourg, France*

E-mail: max.koegler@tu-ilmenau.de

S1 Tip termination

The attachment of a single Nc molecule to the STM tip apex followed a previously reported procedure.[1] The tip was positioned above the center of an individual Nc molecule anchored at a Cu(111) surface defect. The feedback loop was then disabled ($-40\,\mathrm{mV}$, $20\,\mathrm{pA}$) and the tip approached by $350\,\mathrm{pm}$ at $-1\,\mathrm{mV}$. The successful transfer of Nc from the surface to the tip was verified by IETS of the Nc spin excitation on clean Cu(111). Additionally, the Nc-decorated tip apex was imaged by scanning across a single Cu atom that had been deposited prior to tip termination. A similar approach to the spatial characterization of the tip apex was applied before.[2–4]

S2 Tilt angle of Nc-decorated tip

As shown in the main text, an Nc-terminated tip gives rise to a ringlike pattern in the STM image of a single adsorbed Cu atom with nonunifom contrast (Fig. 1d). The maximum apparent height difference Δh observed in the ring is assigned to the tilted adsorption of Nc at the tip. The tilt angle α subtending the surface normal can be estimated from $\alpha = \tan^{-1}(\Delta h/\Delta x)$, where Δx is the lateral distance spanning the minimum and maximum apparent height of the ring. The tips used in the present experiments exhibited $\alpha \approx 6°$, which is in agreement with previous findings.[5,6]

S3 Fabrication of molecule-cluster assemblies

In the first step of the manipulation experiments, a single Cu atom is transferred from the tip to the surface (Fig. S1a) according to a previously reported method.[7] In the second step, one of the three deposited Cu atoms is dragged to another Cu atom to form a Cu_2 cluster (Fig. S1b). To this end, the metal tip is approached to the single Cu atom until the junction conductance reaches $\approx G_0/8$ at 10 mV.[8] The resulting Cu dimer is unstable at the temperature of the experiments and can be easily distorted by the imaging process (Fig. S1b), which agrees with previous findings.[9,10] Subsequently, the remaining Cu atom is dragged close to the Cu_2 cluster and a Cu_3 chain forms (Fig. S1c). The linear atomic assembly is preferred to the compact arrangement, which likewise is in accordance with earlier results.[9,10] The chain is oriented along a compact Cu(111) lattice direction and individual Cu atoms are adsorbed on face-centered cubic sites.[9,10]

After the successful cluster assembly, the tip is decorated with a single Nc molecule, as described in Sec. S1. The subsequent attachment of Nc to the pristine Cu(111) surface, the Cu adatom, and the Cu_3 cluster proceeds via the approach of the Nc-terminated tip. For Cu(111), the feedback loop was deactivated at −40 mV, 20 pA, for the Cu adatom and the Cu_3 cluster at 10 mV, 20 pA prior to approaching the tip by 450 pm at 50 mV (surface),

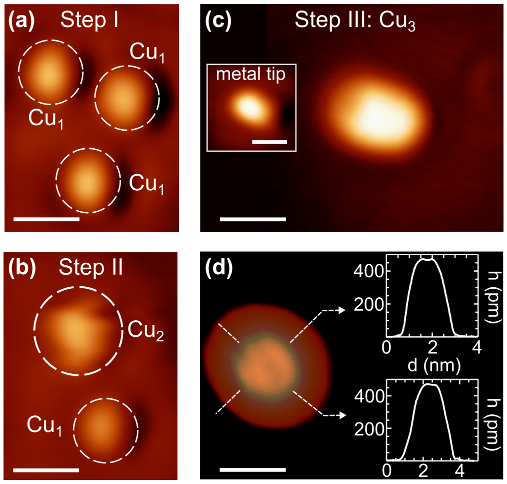

Figure S1: Fabrication of Nc-cluster assemblies. (a) STM image of three individual Cu atoms on Cu(111) acquired with a metal tip (80 mV, 20 pA). (b) As (a) after Cu_2 assembly. (c) STM image of a linear Cu_3 cluster on Cu(111) acquired with an Nc tip (−200 mV, 20 pA). Inset: metal-tip STM image of the Cu_3 cluster in (c) (80 mV, 20 pA). (d) STM image of Nc-Cu_3 recorded with a metal tip (80 mV, 20 pA). Insets: cross-sectional profiles acquired atop Nc-Cu_3 along the dashed lines. The scale bars in all STM images indicate 1 nm.

200 pm (adatom) and 250 pm (Cu_3) at 10 mV. These data were found to scatter, which hints at the dependence of the assembly on the actual tip.

The Nc-adatom compound can easily be distorted by imaging (inset to Fig. 2a of the article), in agreement with observations from Nc-adatom compounds on Cu(100).[11] One stable junction was achieved and reproducible dI/dV spectra of the spin excitation gap could be acquired, that is, two consecutive measurements, each performed after a control image yielded the same $D = 4.14$ meV.

The Nc-Cu_3 assembly is stable (Fig. S1d). Cross-sectional profiles (insets to Fig. S1d) show that Nc is slightly tilted along the long axis of the Cu_3 chain. The stability allowed spectroscopic data acquisition from the tunneling to the contact range of tip separations. Spectroscopy was performed for one representative Nc-Cu_3. At a given tip–surface distance in the tunneling range, a reproducible $D = 3.31$ meV resulted from three of such measurements. The associated standard deviation of only 0.01 meV demonstrates the low scattering of the spectra.

S4 Junction relaxations

Two observations suggest that relaxation effects of the junction geometry play a minor role in the changes of the spin excitation energy above Cu(111). First, the variation of $D = D(J\varrho)$

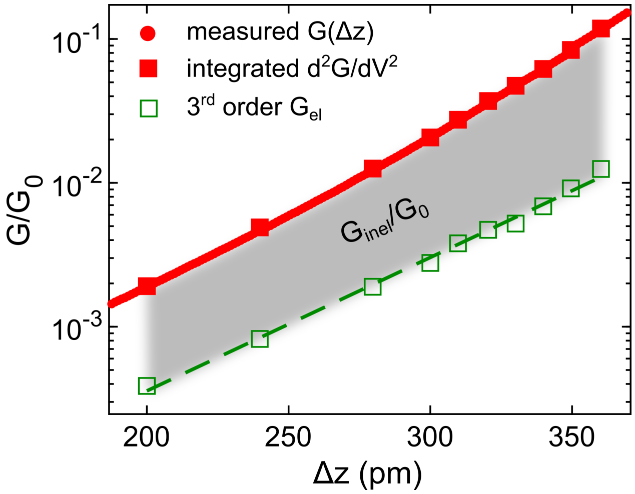

Figure S2: Variation of junction conductance G with Nc tip excursion Δz on Cu(111) at 10 mV. Measured $G(\Delta z)$ appears as dots (top graph), while integrated $\mathrm{d}^2I/\mathrm{d}V^2$ data (divided by 10 mV) are depicted as solid squares (integration interval: $0 \leq V \leq 10\,\mathrm{mV}$). Elastic contribution G_{el} (open squares) to G results from matching $\mathrm{d}^2I/\mathrm{d}V^2$ data within the third-order model. The green dashed line depicts an exponential fit to G_{el} data. The shaded area illustrates the inelastic contribution G_{inel} to G.

matches previous calculations (Fig. 4d) where mechanical deformations of the magnetic impurity are absent.[12–14] Second, deviations of $G = G(\Delta z)$ (Fig. 4b) from a uniform exponential variation, which are often assigned to junction relaxations, can readily be explained by the shift of the spin excitation onset to lower voltages in $\mathrm{d}I/\mathrm{d}V$ spectra. To see this, Fig. S2 compares the measured $G(\Delta z)$ (dots) with numerically integrated $\mathrm{d}^2G/\mathrm{d}V^2$ spectroscopic data (solid squares). Both graphs match very well. In order to rule out elastic effects from contributing to the shifted spectroscopic signature, the elastic component G_{el} of G resulting from σ_{el} in the third-order scattering model is added to Fig. S2 (open squares). Obviously, G_{el} virtually follows a uniform exponential variation, that is, elastic effects do not contribute to the buckled evolution of $G(\Delta z)$.

S5 Dynamical scattering models

Second order. A first approach to model the Nc spin excitation spectrum considers the isolated exchange interaction between the spin $\mathbf{s}$ of the tunneling electron and the molecular spin $\mathbf{S}$. The scattering potential is proportional to $\mathbf{s}\cdot\mathbf{S}$. Writing the differential conductance as $\mathrm{d}I/\mathrm{d}V \equiv \sigma = \sigma_{\mathrm{el}} + \sigma_{\mathrm{inel}}$ with elastic (el) as well as inelastic (inel) contributions and applying Fermi's Golden Rule to the aforementioned scattering process leads to in second order[15]

$$\sigma_{\mathrm{inel}} \equiv \sigma_{\mathrm{inel}}^{(2)} = \sigma_0 \sum_{if} p_i \left|\mathbf{M}_{if}\right|^2 \Xi(\mathrm{e}V - \varepsilon_{if}, T) \tag{S1}$$

($|i\rangle$,$|f\rangle$: all possible initial and final spin states of Nc, p_i: initial-state occupation, $\mathbf{M}_{if}$: transition matrix element, ε_{if}: energy difference of specific initial and final spin states). The temperature-broadened step function Ξ reads[16]

$$\Xi(\mathrm{e}V - \varepsilon_{if}, T) = \frac{\exp(v)\cdot[\exp(v) - v - 1]}{[\exp(v) - 1]^2} \tag{S2}$$

with $v = (\mathrm{e}V - \varepsilon_{if})/(\mathrm{k_B}T)$ and $\mathrm{k_B}$ the Boltzmann constant. The modulation broadening[16,17] of $\mathrm{d}I/\mathrm{d}V$ data is negligible compared with the temperature broadening for the modulation voltages used in the experiments. The matching of experimental $\mathrm{d}^2I/\mathrm{d}V^2$ data (Fig. S3a) proceeds via the numerical derivative $\mathrm{d}\sigma/\mathrm{d}V$ within the second-order scattering model.

For sufficiently small currents, nonequilibrium population of spin states is suppressed and p_i solely reflects the thermal occupation of the spin states, which at the experimental temperature $T = 5\,\mathrm{K}$ is the ground state $|M_S = 0\rangle$ since $\mathrm{k_B}T \ll D$. The matrix elements $\mathbf{M}_{if}$ for exchange scattering between the spin states of the scattering electron $(\varphi_{i'}, \varphi_{f'})$ and Nc (ψ_i, ψ_f) are defined as

$$\mathbf{M}_{if} = \sum_{i',f'} \langle \varphi_{f'}, \psi_f | \hat{\mathbf{s}} \cdot \hat{\mathbf{S}} | \psi_i, \varphi_{i'} \rangle \tag{S3}$$

with $\hat{\mathbf{s}}$ and $\hat{\mathbf{S}}$ the associated spin operators. For the transition $|i\rangle \equiv |M_S = 0\rangle \rightarrow |f\rangle \equiv |M_S = \pm 1\rangle$, the nonzero squared matrix elements are calculated as $|\mathbf{M}_{0,+1}|^2 = 1/2 = |\mathbf{M}_{0,-1}|^2$

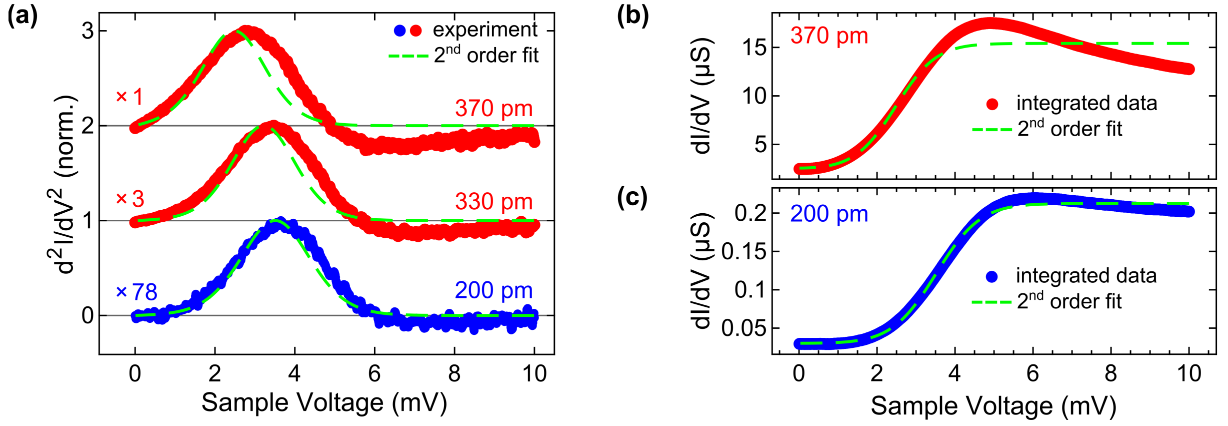

Figure S3: Failure of the second-order model. (a) Spectra of $\mathrm{d}^2I/\mathrm{d}V^2$ (dots) acquired with an Nc tip atop Cu(111) at the indicated tip excursions and normalized to the respective maxima (reference feedback loop parameters: 10 mV, 20 pA). Fits to the data are depicted as dashed lines. The spectra and fits are vertically offset with the horizontal line marking $\mathrm{d}^2I/\mathrm{d}V^2 = 0$ for each spectrum. (b) Numerically integrated data of (a) at $\Delta z = 370$ pm. (c) Numerically integrated data of (a) at $\Delta z = 200$ pm.

with $\varepsilon_{0,+1} = D = \varepsilon_{0,-1}$ giving rise to $f(V)$ introduced in the main text ($\hbar = 1$). The temperature T and the spin excitation energy D served as fit parameters. The resulting temperature reliably reproduced the experimental value of $T = 5$ K.

In the far tunneling range of tip–surface distances, the second-order model is successful (Fig. 1e in the main text), while it fails in describing the data acquired at small tip–surface separations. This failure is illustrated in Fig. S3a for fits to $\mathrm{d}^2I/\mathrm{d}V^2$ data at different tip–surface distances and in Fig. S3b,c for the $\mathrm{d}I/\mathrm{d}V$ signal. The main deviation occurs at the high-voltage tails. The shallow indentation following the peak in $\mathrm{d}^2I/\mathrm{d}V^2$ (Fig. S3a), which is associated with the cusps in $\mathrm{d}I/\mathrm{d}V$ (Fig. S3b,c) is characteristic for third-order tunneling processes explained in the next paragraph.

Third order. In the third-order scattering model, the Kondo exchange interaction $-J\varrho\,\tilde{\mathbf{s}}\cdot\mathbf{S}$ is introduced where the spin of substrate electrons ($\tilde{\mathbf{s}}$) enters into the scattering potential (J: Kondo magnetic exchange energy, ϱ: density of electron states at the Fermi energy (E_F)).[15,18,19] Here, processes are included where, e. g., substrate electrons scatter the Nc

spin into some virtual intermediate state before the tunneling electron transfers this state to the final state. Considering scattering processes up to third-order is associated with the following transition matrix element that couples initial (i) and final (f) spin states of Nc via intermediate (m) spin states[18,19]

$$\left|\mathbf{M}_{if}^{(3)}\right|^2 = \left|\mathbf{M}_{if}\right|^2 + J\rho \sum_m \int \frac{2\Re\left(\mathbf{M}_{mi}\mathbf{M}_{fm}\mathbf{M}_{if}\right)}{\tilde{\varepsilon}_{im'} + \varepsilon_{im}}\,\mathrm{d}\tilde{\varepsilon}_{m'} \tag{S4}$$

The sum in eq S4 reflects discrete Nc spin states, while the integral considers the continuum of nearly-free substrate electron states with energy $\tilde{\varepsilon}_{m'}$. Evaluating the transition matrix elements in the numerator of eq S4 by using eq S3 yields $\Re\left(\mathbf{M}_{mi}\mathbf{M}_{fm}\mathbf{M}_{if}\right) = -1/4$ as the only nonzero contribution for the transitions $|i\rangle \equiv |M_S = 0\rangle \to |m\rangle \equiv |M_S = 1\rangle \to |f\rangle \equiv |M_S = 1\rangle$ and $|i\rangle \equiv |M_S = 0\rangle \to |m\rangle \equiv |M_S = -1\rangle \to |f\rangle \equiv |M_S = -1\rangle$.

In analogy to eq S1, the inelastic differential conductance in the third-order scattering model is expressed as

$$\sigma_{\text{inel}} = \sigma_0 \sum_{if} p_i \left|\mathbf{M}_{if}^{(3)}\right|^2 \Xi(\mathrm{e}V - \varepsilon_{if}, T) = \sigma_{\text{inel}}^{(2)} + \sigma_{\text{inel}}^{(3)} \tag{S5}$$

Taking further into account the Fermi-Dirac occupation statistics $F(\tilde{\varepsilon}) = 1/\{1 + \exp[(\tilde{\varepsilon} - E_{\mathrm{F}})/(\mathrm{k_B}T)]\}$ for initial, intermediate and final states, $\sigma_{\text{inel}}^{(3)}$ can be recast to give

$$\sigma_{\text{inel}}^{(3)} = -1 \cdot \sigma_0 \cdot J\rho \cdot \left[g(\mathrm{e}V - D) + g(\mathrm{e}V + D)\right] \cdot \left[\Xi(\mathrm{e}V - D, T) + \Xi(-\mathrm{e}V - D, T)\right] \tag{S6}$$

with

$$g(\tilde{\varepsilon}) = \int_{-\infty}^{\infty} \int_{-\hbar\omega_0}^{\hbar\omega_0} \frac{1 - F(\tilde{\varepsilon}_{m'}, T)}{\tilde{\varepsilon}_{m'} - \tilde{\varepsilon}_i + \mathrm{i}\Gamma_0} F'(\tilde{\varepsilon}_i - \tilde{\varepsilon}, T)\,\mathrm{d}\tilde{\varepsilon}_{m'}\,\mathrm{d}\tilde{\varepsilon}_i \tag{S7}$$

($\mathrm{i}^2 = -1$, Γ_0: electron lifetime broadening) where F' denotes the derivative of the Fermi-Dirac function with respect to $\tilde{\varepsilon}$.[14,18,20] The inner integration is restricted to $-\hbar\omega_0 \leq \tilde{\varepsilon}_{m'} - E_{\mathrm{F}} \leq \hbar\omega_0$, which reflects the bandwidth of relevant substrate electron states.[18,19]

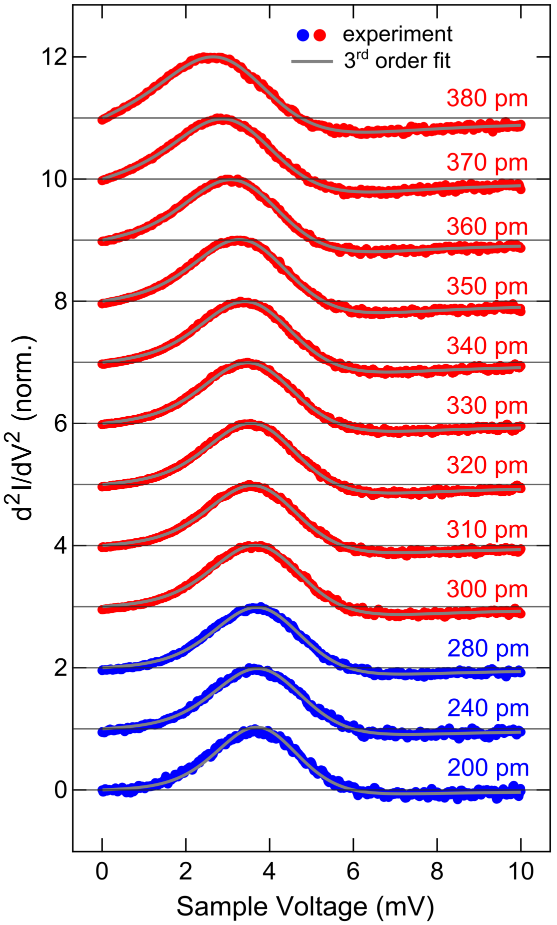

Figure S4: Successful description of spectroscopic data within the third-order model. Spectra of $\mathrm{d}^2I/\mathrm{d}V^2$ (dots) acquired with an Nc tip atop Cu(111) and normalized by the respective maximum at the indicated tip excursions Δz. Increasing Δz corresponds to decreasing tip–sample distance. The spectra are vertically offset with the horizontal lines marking $\mathrm{d}^2I/\mathrm{d}V^2 = 0$ for each tip excursion. Feedback loop parameters defining $\Delta z = 0$: 10 mV, 20 pA.

The fit of the resulting $\sigma = \sigma_{\mathrm{el}} + \sigma^{(2)}_{\mathrm{inel}} + \sigma^{(3)}_{\mathrm{inel}}$ to the experimental $\mathrm{d}I/\mathrm{d}V$ data[15] enables the extraction of $J\varrho$ and D, which served as fit parameters. To this end, experimental $\mathrm{d}^2I/\mathrm{d}V^2$ data were corrected for offsets and then numerically integrated and symmetrized with respect to zero sample voltage. The parameters Γ_0 and $\hbar\omega_0$ turned out to be dispensable for the overall quality of the fit and were therefore set to $\Gamma_0 = 5\,\mu\mathrm{eV}$ and $\hbar\omega_0 = 20\,\mathrm{meV}$.[14,15] The fits reproduce the spectroscopic data for all measured tip–surface distances (Fig. S4).

S6 Kondo exchange renormalization

The measured spin excitation spectrum of Nc on Cu(111) reflects spin excitation energies D that are renormalized due to the interaction of the Nc spin with the substrate electron continuum. This Kondo exchange interaction was previously shown[12–14] to change the bare Nc spin energy levels according to $\varepsilon_\alpha(J\varrho) = \varepsilon_\alpha(0) + \delta\varepsilon_\alpha(J\varrho)$ with:[14]

$$\delta\varepsilon_\alpha(J\varrho) \approx (J\varrho)^2 \sum_{n\neq\alpha} \sum_{n',\alpha'} \frac{\left|\langle \varphi_{n'}, \psi_n | \hat{\tilde{\mathbf{s}}} \cdot \hat{\mathbf{S}} | \psi_\alpha, \varphi_{\alpha'} \rangle\right|^2}{\varepsilon_\alpha - \varepsilon_n + \tilde{\varepsilon}_{\alpha'} - \tilde{\varepsilon}_{n'}} \tag{S8}$$

From eq S8, it can be inferred that the Nc spin states $|\alpha\rangle \equiv |M_S = \pm 1, 0\rangle$ move more closely in energy. Consequently, the experimentally measured $D = \varepsilon_{0,\pm 1}$ is lowered. Moreover, eq S8 predicts the approximate behavior $D(J\varrho) \approx D_0 - c(J\varrho)^2$ with $D_0 = D(0)$ the spin excitation energy in the absence of Kondo exchange coupling and a constant c. Since D and $J\varrho$ are extracted from the matching procedure, this prediction can be tested.

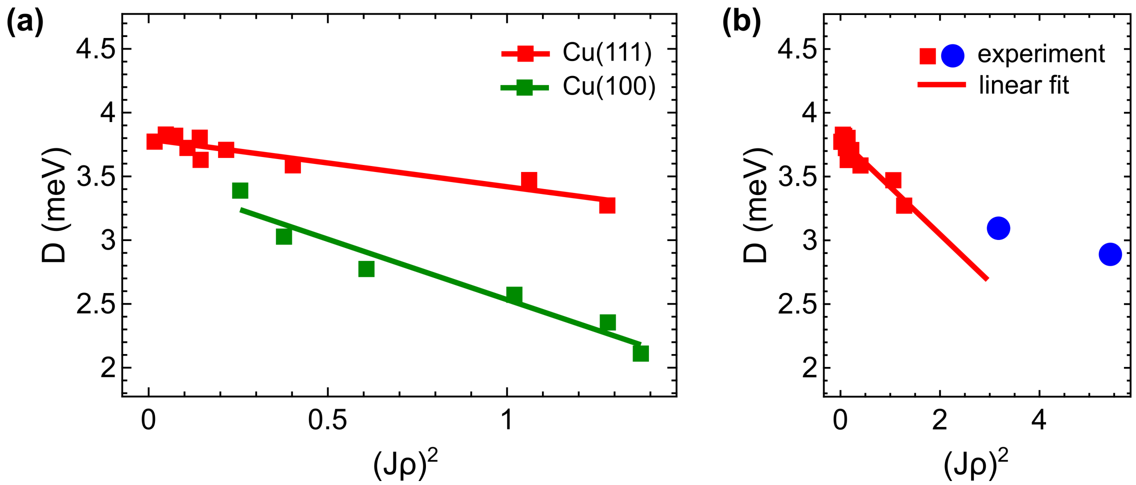

Figure S5: Variation of D with $(J\varrho)^2$ for an Nc tip on Cu(111) (this work) and Cu(100).[11] (a) Green and red squares depict D extracted from matching spectroscopic data within the third-order model while solid lines are linear fits to the data. (b) Plot of Cu(111) data for an extended range of $J\varrho$ revealing the deviation (dots) from the predicted behavior $D(J\rho)$. The solid line is an extrapolated linear fit to the data presented in (a) (red squares). The additional data were extracted from spectroscopic data acquired at tip excursions 370 pm and 380 pm.

Figure S5 shows a plot of D as a function of $(J\varrho)^2$. For sufficiently low $J\varrho$, D indeed exhibits the predicted linear variation with $(J\varrho)^2$ (Fig. S5a). By courtesy of the Kiel group, experimental spin excitation spectroscopic data were provided for an Nc tip on Cu(100).[11] Describing these data within the third-order scattering model likewise gives rise to a linear evolution of D with $(J\varrho)^2$, albeit with a different slope. While for Cu(111), $c = 0.37\,\text{meV}$ was obtained, the slope for the Cu(100) data is $c = 1.00\,\text{meV}$. A clear-cut rationale for this observation is missing to date. However, the slope c is determined by the bandwidth $\hbar\omega_0$ of the substrate electrons Nc is coupled to.[13,14] Therefore, the different electronic structure of Cu(111) and Cu(100) may contribute to the deviations in c. The different values for D_0, i. e., $D_0 = 3.8\,\text{meV}$ for Cu(111) and $D_0 = 3.5\,\text{meV}$ for Cu(100), are likely associated with the different tip shapes used in the separate experiments (Fig. 2 of the main text).

Figure S5b shows that for larger values of $J\varrho$ deviations from the predicted behavior of $D(J\varrho)$ occur, which reflect the limitations of the perturbation theory underlying the derivation of $\delta\varepsilon_\alpha$ (eq S8). Indeed, the two additional data points in Fig. S5b were obtained for tip excursions $\Delta z = 370\,\text{pm}$ and $\Delta z = 380\,\text{pm}$, which are close to the collapse of the tunneling barrier (Fig. 3b of the main text) and entailed by an increased interaction between the Nc tip and the surface.

S7 Third-order analysis applied to Nc tips in the far tunneling range

Figure 2d of the article presents the collection of spin excitation energies D obtained from ten different Nc-terminated tips in the far tunneling range above pristine Cu(111) (feedback loop parameters for spectroscopy: 10 mV, 30 pA–100 pA). The $\mathrm{d}I/\mathrm{d}V$ spectrum of the lower inset to Fig. 2d is repeated here (Fig. S6a). Superimposed with the experimental data (dots) are fits within the second-order (dashed line) and third-order (solid line) scattering model (see Sec. S5). Obviously, the latter provides an improved match compared with the

former because the cusps at $\approx \pm 6\,\mathrm{mV}$ are well reproduced for $J\varrho = -0.15 \pm 0.01$. Indeed, the reliability factors R^2 of the least-mean-squares fits are $R^2 = 0.995$ (second order) and $R^2 = 0.998$ (third order). Importantly, the third-order contributions ($\propto |J\varrho|$, eq S6) signaled by cusps are very weak over all tested Nc tips (Fig. S6b) and thus, the extraction of D via the second-order fit to tunneling spectroscopic data as done for Fig. 2d of the article is justified.

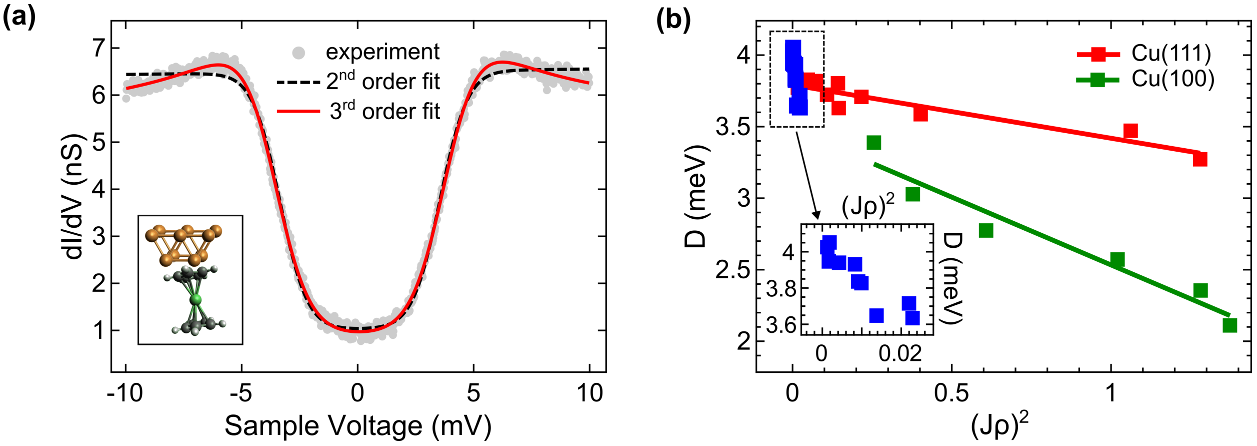

Figure S6: Third-order analysis for tunneling spectra acquired with different Nc tips. (a) Spectrum of dI/dV (dots) of an Nc-terminated tip above Cu(111) (feedback loop parameters: $-10\,\mathrm{mV}$, $50\,\mathrm{pA}$) together with second-order (dashed line) and third-order (solid line) fits. (b) Comparison of D-versus-$(J\varrho)^2$ data (squares) extracted from third-order fits to dI/dV spin excitation spectra of 10 Nc-terminated tips (blue) with results obtained from Nc tips approaching Cu(111) (red) and Cu(100) (green). Solid lines are linear fits to the data. Inset: close-up view of (b) for data obtained with the ten tips in the far tunneling range.

Because of the applicability of the third-order model to tunneling spectra of Nc tips it is tempting to assume Kondo exchange coupling of Nc with the tip it is attached to as a rationale to their wide D variation. To further explore this assumption, the D-versus-$(J\varrho)^2$ behavior was extracted for all tips of Fig. 2d via third-order fits. Since, to first order, the Nc–tip coupling is conceptually similar to approaching an Nc tip to Cu(111) or Cu(100), a similar D variation is expected. In fact, Fig. S6b (blue squares) shows a strong variation of D across the 10 tips for nearly vanishing $J\varrho$. A linear fit to these data yields a slope of $-17\,\mathrm{meV}$, which exceeds the slope observed for approaching an Nc tip to Cu(111) (red squares) and Cu(100) (green squares) by more than an order of magnitude (see Sec. S6). Therefore, the scattering of D is most likely not driven by the Kondo exchange interaction. Rather, it is

surmised that Kondo exchange coupling is dominated by Nc–tip charge transfer,[1] which may depend on the actual tip geometry.

References

(1) Ormaza, M.; Bachellier, N.; Faraggi, M. N.; Verlhac, B.; Abufager, P.; Ohresser, P.; Joly, L.; Romeo, M.; Scheurer, F.; Bocquet, M.-L.; Lorente, N.; Limot, L. Efficient Spin-Flip Excitation of a Nickelocene Molecule. *Nano Letters* **2017**, *17*, 1877–1882.

(2) Kelly, K. F.; Sarkar, D.; Prato, S.; Resh, J. S.; Hale, G. D.; Halas, N. J. Direct observation of fullerene-adsorbed tips by scanning tunneling microscopy. *J. Vac. Sci. Technol. B* **1996**, *14*, 593–596.

(3) Schull, G.; Frederiksen, T.; Arnau, A.; Sanchez-Portal, D.; Berndt, R. Atomic-scale engineering of electrodes for single-molecule contacts. *Nat. Nanotechnol.* **2011**, *6*, 23–27.

(4) Brand, J.; Leitherer, S.; Papior, N. R.; Néel, N.; Lei, Y.; Brandbyge, M.; Kröger, J. Nonequilibrium Bond Forces in Single-Molecule Junctions. *Nano Letters* **2019**, *19*, 7845–7851.

(5) Czap, G.; Wagner, P. J.; Xue, F.; Gu, L.; Li, J.; Yao, J.; Wu, R.; Ho, W. Probing and imaging spin interactions with a magnetic single-molecule sensor. *Science* **2019**, *364*, 670–673.

(6) Verlhac, B.; Bachellier, N.; Garnier, L.; Ormaza, M.; Abufager, P.; Robles, R.; Bocquet, M.-L.; Ternes, M.; Lorente, N.; Limot, L. Atomic-scale spin sensing with a single molecule at the apex of a scanning tunneling microscope. *Science* **2019**, *366*, 623–627.

(7) Limot, L.; Kröger, J.; Berndt, R.; Garcia-Lekue, A.; Hofer, W. A. Atom Transfer and Single-Adatom Contacts. *Phys. Rev. Lett.* **2005**, *94*, 126102.

(8) Ternes, M.; Lutz, C. P.; Hirjibehedin, C. F.; Giessibl, F. J.; Heinrich, A. J. The Force Needed to Move an Atom on a Surface. *Science* **2008**, *319*, 1066–1069.

(9) Repp, J.; Meyer, G.; Rieder, K.-H.; Hyldgaard, P. Site Determination and Thermally Assisted Tunneling in Homogenous Nucleation. *Phys. Rev. Lett.* **2003**, *91*, 206102.

(10) Fölsch, S.; Hyldgaard, P.; Koch, R.; Ploog, K. H. Quantum Confinement in Monatomic Cu Chains on Cu(111). *Phys. Rev. Lett.* **2004**, *92*, 056803.

(11) Mohr, M.; Gruber, M.; Weismann, A.; Jacob, D.; Abufager, P.; Lorente, N.; Berndt, R. Spin dependent transmission of nickelocene-Cu contacts probed with shot noise. *Physical Review B* **2020**, *101*, 075414.

(12) Cohen-Tannoudji, C.; Dupont-Roc, J.; Grynberg, G. *Atom-photon interactions: Basic processes and applications*; Wiley science paperback series; Wiley: New York, NY, 2008.

(13) Oberg, J. C.; Calvo, M. R.; Delgado, F.; Moro-Lagares, M.; Serrate, D.; Jacob, D.; Fernández-Rossier, J.; Hirjibehedin, C. F. Control of single-spin magnetic anisotropy by exchange coupling. *Nature Nanotechnology* **2014**, *9*, 64 – 68.

(14) Jacobson, P.; Herden, T.; Muenks, M.; Laskin, G.; Brovko, O.; Stepanyuk, V.; Ternes, M.; Kern, K. Quantum engineering of spin and anisotropy in magnetic molecular junctions. *Nature Communications* **2015**, *6*, 8536.

(15) Ternes, M. Spin excitations and correlations in scanning tunneling spectroscopy. *New Journal of Physics* **2015**, *17*, 063016.

(16) Lambe, J.; Jaklevic, R. C. Molecular Vibration Spectra by Inelastic Electron Tunneling. *Phys. Rev.* **1968**, *165*, 821–832.

(17) Klein, J.; Léger, A.; Belin, M.; Défourneau, D.; Sangster, M. J. L. Inelastic-Electron-Tunneling Spectroscopy of Metal-Insulator-Metal Junctions. *Phys. Rev. B* **1973**, *7*, 2336–2348.

(18) Appelbaum, J. "s-d" Exchange Model of Zero-Bias Tunneling Anomalies. *Phys. Rev. Lett.* **1966**, *17*, 91–95.

(19) Appelbaum, J. A. Exchange Model of Zero-Bias Tunneling Anomalies. *Phys. Rev.* **1967**, *154*, 633–643.

(20) Ternes, M. Probing magnetic excitations and correlations in single and coupled spin systems with scanning tunneling spectroscopy. *Progress in Surface Science* **2017**, *92*, 83 – 115.
